# *De-novo* RNA Sequencing and Metabolite Profiling to Identify Genes Involved in Anthocyanin Biosynthesis in Korean Black Raspberry (*Rubus coreanus* Miquel)

**DOI:** 10.1371/journal.pone.0088292

**Published:** 2014-02-05

**Authors:** Tae Kyung Hyun, Sarah Lee, Yeonggil Rim, Ritesh Kumar, Xiao Han, Sang Yeol Lee, Choong Hwan Lee, Jae-Yean Kim

**Affiliations:** 1 Division of Applied Life Science (BK21plus), Plant Molecular Biology and Biotechnology Research Center, Gyeongsang National University, Jinju, Republic of Korea; 2 Division of Bioscience and Biotechnology, Konkuk University, Seoul, Republic of Korea; The Centre for Research and Technology, Hellas, Greece

## Abstract

The Korean black raspberry (*Rubus coreanus* Miquel, KB) on ripening is usually consumed as fresh fruit, whereas the unripe KB has been widely used as a source of traditional herbal medicine. Such a stage specific utilization of KB has been assumed due to the changing metabolite profile during fruit ripening process, but so far molecular and biochemical changes during its fruit maturation are poorly understood. To analyze biochemical changes during fruit ripening process at molecular level, firstly, we have sequenced, assembled, and annotated the transcriptome of KB fruits. Over 4.86 Gb of normalized cDNA prepared from fruits was sequenced using Illumina HiSeq™ 2000, and assembled into 43,723 unigenes. Secondly, we have reported that alterations in anthocyanins and proanthocyanidins are the major factors facilitating variations in these stages of fruits. In addition, up-regulation of *F3′H1*, *DFR4* and *LDOX1* resulted in the accumulation of cyanidin derivatives during the ripening process of KB, indicating the positive relationship between the expression of anthocyanin biosynthetic genes and the anthocyanin accumulation. Furthermore, the ability of RcM*CHI2* (*R. coreanus* Miquel chalcone flavanone isomerase 2) gene to complement *Arabidopsis transparent testa 5* mutant supported the feasibility of our transcriptome library to provide the gene resources for improving plant nutrition and pigmentation. Taken together, these datasets obtained from transcriptome library and metabolic profiling would be helpful to define the gene-metabolite relationships in this non-model plant.

## Introduction

*Rubus* is one of the most diverse and the largest genera in the *Rosaceae* family, and encloses around 600 to 800 species including blackberries, raspberries and their hybrids [1], [2]. Although the recent use of their fruits as fresh and processed food items such as jams, jellies and juices represents a multimillion-dollar industry [2], in addition, it also attracts international market due to its medicinal properties, including anti-inflammatory effect on ulcers [3], the reduction of blood cholesterol levels [4] and cell proliferation [5] in animal models. The Korean black raspberry (KB) is the fruit of *Rubus coreanus* Miquel, which is cultivated in Southeast Asia [6]. For several decades, KB has been used as traditional herbal medicine for the treatment of spermatorrea, enuresis and asthma [6]. Presence of flavonoids, anthocyanins, tannins, triterpenoids, phenolic acids and organic acids in its extract have been reported to exhibit many medicinal properties such as anti-oxidant [7], anti-inflammatory [6], immune modulation [8] and anti-cancer activities [9]. Interestingly, the unripe KB has been widely used as traditional herbal medicine, whereas ripe KB is generally used as a processed food [10], [11]. This finding indicates that the biochemical composition of KB might be strongly influenced by developmental changes during ripening process. However, the alteration of the biochemical composition and the biosynthetic pathways during KB ripening remains poorly understood due to the lack of large-scale genomics and metabolomics information.

The fruit ripening is a genetically programmed process that involves a number of biochemical and physiological processes assisted by variations in gene expression and enzyme activities. This process generally includes the modification of the structure and composition of cell wall polysaccharides, the conversion of starch to sugars, the degradation of chlorophyll, the biosynthesis of pigments as well as the accumulation of flavor and aromatic volatiles [12], [13]. These various biochemical reactions hint towards the dramatic changes in the complex network of metabolites mediated by changes in gene expression and enzyme activities during fruit ripening process. In this regard, transcriptomics and metabolomics together provide a unique dissection tool for the better understanding of a biological system due to their ability to follow a relatively large number of genes and compounds. So far, metabolite profiling has been successfully used for analyzing metabolic networks during fruit ripening of various plant species, including strawberry [13], [14], tomato [15], [16], peach [17], blueberry [18], sweet cherry [19] and grape [20]. In case of berries and grapes, the accumulation of anthocyanins contributes to quality characteristics expected from a ripe fruit. The production of anthocyanin during the fruit ripening process is an essential trait for attracting fruit-eating animals and hence dispersal of seeds [18], [21]. For instance, at the early stages of bilberry development, procyanidins (proanthocyanidin) and quercetin (flavonol) are the major flavonoids. Therefore, anthocyanin has been suggested as a marker of ripening. The increased antioxidant capacity by the enrichment of anthocyanin delays the over ripening, and results in extending shelf life of tomato [22]. In addition, anthocyanins play an important role in limiting the spread of fungal infection [22], indicating that anthocyanins are not just pigments but also act as functional phytochemicals. The production and distribution of anthocyanin are governed by metabolic networks regulated by genetic, developmental and environmental conditions [23], and are strongly correlated with the expression of flavonoid pathway genes. The early pathway genes, required for chalcones and flavonols, are controlled by pathway-specific MYB transcription factors [24], whereas the regulation of the pathways leading to anthocyanins is mediated by the combination and interaction of R2R3-MYB proteins, basic helix-loop-helix (bHLH) proteins and WD-repeat proteins [25]. Although above studies indicate that the integrative comparative analysis of transcriptomics and metabolomics gives important insights into gene-regulatory and metabolic events associated with fruit ripening processes, this approach has been limited to the sequenced plants.

Next-generation sequencing technology (NGS) has been proven to be a powerful and cost-effective tool for the genome sequencing, genome re-sequencing, miRNA expression profiling and DNA methylation analysis [26], [27]. Recently, *de-novo* transcriptome sequencing has been widely used for functional gene discovery in non-model organisms that lack reference genome information [28]–[30]. In addition, this approach has also allowed the analysis of gene expression profiling using quantitative (q) PCR, microarrays or the quantification of short cDNA reads [29]–[31].

To analyze the key components of the biochemical pathways involved during KB ripening processes, we have employed the RNA-Seq method using the Illumina HiSeq™ 2000 to generate large amount of sequenced transcriptome of KB. We have generated 54,083,500 sequence reads that were assembled into 43,723 unigenes. Metabolite profiling using gas chromatography-ion trap-mass spectrometry (GC-IT-MS) and ultra performance liquid chromatography-quadrupole-time of flight-mass spectrometry (UPLC-Q-TOF-MS) showed the first high-resolution picture of the metabolic dynamics during KB ripening and suggested that anthocyanin is a highly enriched flavonoid in the later stages of the ripening process. To further validate the findings, expression analysis of anthocyanin biosynthesis genes in different ripening stages was carried out. The assembled, annotated transcriptome and metabolite profiling would provide an invaluable resource for better understanding of the ripening process as well as functional genomics in *R. coreanus* Miquel in future.

## Materials and Methods

### Sample collection and RNA isolation

KB (*R. coreanus* Miquel) plants were cultivated in Gochang Black Raspberry Research Institute, Republic of Korea. Fruit samples (almost 50 fruits of the same maturation degree) were collected from 5 to 10 individual plants. The fruit sampling includes three different ripening stages, the orange-turning fruit [15 days post-anthesis (15 DPA), stage 1], red fruit (20 DPA, stage 2) and dark red fruit (25 DPA, stage 3). After harvesting, samples were immediately frozen in liquid nitrogen and kept at −80°C until use for sample preparation.

Total RNA was isolated from stage 2 fruits using RNEasy Plant Mini kit (Qiagen, USA) according to the manufacturer's instructions. 50 mg of ground samples was added to the extraction buffer (RLC buffer) containing 2% beta-mercaptoethanol. The cleared lysate was centrifuged in the RNeasy spin column. The spin column membrane was washed using buffer RW1 and buffer RPE. Total RNA was eluted from membrane using 30 ul of RNase-free water. Total RNA was treated with RNase-free DNase I (Promega, USA) to avoid genomic DNA contamination. Quantity and quality (purity and integrity) of total RNA were assessed by Nanodrop 2000C spectrophotometer (Thermo scientific, USA) and Agilent Bioanalyzer 2100 System (Agilent Technologies, California, USA). Figure S1 shows a total RNA gel-like image produced by the Bioanalyzer.

### Transcriptome assembly and annotation

For transcriptome analysis, mRNA from total RNA was enriched by using the oligo(dT) magnetic beads. Then, mRNA was fragmented to short pieces (about 200 bp), which were then used as templates for random hexamer-primed synthesis of first strand cDNA. After synthesizing second strand cDNA using DNA polymerase I and RNase H, paired-end library was synthesized using the Genomic Sample Prep kit (Illumina, USA) according to the manufacturer's instructions. Short fragments were purified by agarose gel electrophoresis after connecting short fragments to sequencing adapters, and enriched by PCR to create the final cDNA library. The cDNA library was sequenced on the Illumina HiSeq™2000 sequencing platform. The Illumina reads generated in this study are available at the website (http://kimjy.gnu.ac.kr/DB_KB.files/slide0004.htm) and NCBI Sequence Read Archive (SRA) with the accession number SRX347804. The raw reads were cleaned by removing adapter sequences, empty reads, low quality reads (with ambiguous sequences ‘N’) and the reads with more than 10% Q<20 bases (those with a base quality less than 20) through the standard Illumina pipeline including the CASSAVA program. Transcriptome *de-novo* assembly was carried out with short reads assembling program, Trinity (assembly parameter, k-mer value = 25, CPU = 25) [32]. The clean reads (90 nt) with a certain overlap length were combined to form contigs, and then the reads were mapped back to contigs using pair-end joining and gap-filling. With paired-end reads, the distance among contigs and the contigs from the same transcript were determined. Finally, unigenes were generated with zero N values (no unknown bases) in the sequence that could not be extended on either end using the Trinity program.

The unigenes were identified by sequence similarity comparison against NCBI non-redundant protein (NR) database (http://www.ncbi.nlm.nih.gov), Swiss-Prot protein database (http://www.expasy.ch/sprot), the Kyoto Encyclopedia of Genes and Genomes (KEGG) pathway database (http://www.genome.jp/kegg) and Cluster of Orthologous Groups (COG) database (http://www.ncbi.nlm.nih.gov/COG) by applying BLAST with a cut-off E-value of 10^−5^. Blast2GO was used to obtain Gene ontology (GO) annotation of unigenes based on BLASTX hit against NR database with a cut-off E-value of 10^−5^.

From the transcriptome of any species, we can predict its protein sequences which eases in predicting function of the particular gene. Therefore, to analyze similarity between KB transcriptome and other plants, the proteome data sets for *Fragaria vesca*, *Vitis vinifera*, *Manihot esculenta*, *Populus trichocarpa*, *Glycine max*, *Ricinus communis*, *Cucumis sativus*, *Arabidopsis thaliana*, *Arabidopsis lyrata*, *Oryza sativa*, *Sorghum bicolor* and *Zea mays* were downloaded from their respective genome project websites. The KB transcripts (coverage≥70%) were then searched against these proteome sequences using BLASTX program.

### Sample preparation for metabolomic analysis

The frozen fruit samples (∼1 g) from different ripening stages were freeze-dried over 3 days, and homogenized with Mixer Mill (Retsch MM400, Germany) at 30 HZ s^−1^ for 3 min, then stored at below −70°C before extraction. Each sample (10 mg) was extracted with 1 ml methanol using with Mixer Mill (Retsch MM400, Germany) at 30 hertz (HZ) s^−1^ for 5 min, and centrifuged at 4°C and 17000 rpm for 3 min. The supernatant (200 µl) was completely dried using a speed vacuum concentrator (Biotron, Seoul, Korea). For the GC-MS analysis, oximation was carried out by dissolving the dried extracts in 50 µl of methoxyamine hydrochloride (20 mg ml^−1^ in pyridine) and shaking at 30°C for 90 min. Then, samples were silylated with 50 µl of MSTFA at 37°C for 30 min. For UPLC-Q-TOF-MS analysis, dried extracts were resolved with methanol and filtered through a 0.2 µm PTFE filter.

### GC-IT-MS analysis

GC-IT-MS analysis was performed using a Varian CP-3800 GC system equipped with a Varian CP-8400 Autosampler and a Varian 4000 ion trap EI MS detector system (Varian, Palo Alto, CA, USA). 1 µl of each reactant was injected into the GC-IT-MS (with a split ratio of 25∶1) through a VF-1MS capillary column (30 m length×0.25 mm i.d., 0.25 µm film thickness) with helium at a constant flow of 1.0 ml min^−1^ as the carrier gas. The oven temperature was held at 100°C for 2 min, then ramped to 300°C at a rate of 10°C min^−1^ and held for 10 min. The mass data collected in the electron ionization mode with the ionization energy of 70 eV were used for full scan at m/z 50–1,000.

### UPLC-Q-TOF-MS and LC-IT-MS/MS analyses

UPLC was performed on an ACQUITY UPLCTM system (Waters corp., Milford, MA, USA) equipped with a binary solvent delivery system, a UV detector, and an autosampler. 5 µl of each samples was injected into an ACQUITY BEH C18 column (100 mm×2.1 mm i.d., 1.7 µm particle size; Waters, Milford, MA, USA) with a gradient system at a flow rate of 0.3 ml min^−1^. The mobile phases consisted of 0.1% v/v formic acid in water (solvent A) and 0.1% v/v formic acid in acetonitrile (solvent B). Initial condition was 5% solvent B for 1 min, and then linear gradients of 5–100% solvent B from 1 to 10 min. Total run time was 12 min including re-equilibration of the column to the initial conditions. The mass spectrometric data were collected with a Waters Q-TOF Premier (Micromass MS Technologies, Manchester, UK) operated in both negative and positive ion modes with the *m/z* range of 100 to 1000. The source temperature was set at 80°C; the desolvation gas was set to 650 l h^−1^ at a temperature of 300°C; the collision gas was flow 0.3 ml min^−1^ and the collision energy was set at 10 eV. The capillary voltage and sample cone voltage were set at 2.3 kV and 30 V, respectively. The V mode was used for the mass spectrometer and data were collected in the centroid mode with a 0.2 s scan accumulation time. Leucine enkephalin ions were used as the lock mass [*m*/*z* 554.2615 (−) and 556.2771 (+)] at a flow rate of 10 µl min^−1^.

The LC-IT-MS/MS analysis was performed using 212-LC Binary solvent delivery system equipped with a Prostar 410 Autosampler, and a Prostar 335 photodiode array detector (PDA), which was coupled to the Varian 500-MS ion-trap mass spectrometer equipped with an electrospray interface (Varian Tech., Palo Alto, CA, USA). 10 µl of each samples was injected into PurSuit XRs C18 column (100 mm×2.0 mm i.d., 3 µm; Varian, Lake Forest, CA, USA) with a MetaGuard 2.0 PurSuit XRs C18 guard column (Varian, Lake Forest, CA, USA). The column temperature was set at 40°C and flow rate was 0.2 ml min^−1^. The mobile phases consisted of 0.1% v/v formic acid in water (solvent A) and 0.1% v/v formic acid in acetonitrile (solvent B). The gradient applied was as follows: from 0 to 2 min 10% B, from 2 to 10 min to 40% B, from 10 to 20 min 70% B, from 20 to 25 min 90% B, from 25 to 30 min 90% B, from 30.6 min 10% B, and held for 40 min 10% B before the next sample injection. Mass spectra were performed in both negative and positive modes through a range of *m/z* 100 to 1000. The parameters were as follows: drying temperature, 300°C; capillary voltage, 70 V; needle voltage, 5 kV; drying gas pressure (nitrogen), 20 psi; and nebulizer gas pressure (air), 40 psi. MS^n^ analysis was performed using Turbo data-dependent scanning (DDS) under the same conditions used for full scanning. The PDA was set the absorbance from 200 to 600 nm and managed by Polyview 2000 (version 6.9) (Varian, Walnut Creek, CA).

### Data processing and analysis

GC-IT-MS and LC-IT-MS/MS raw data acquired by MS workstation software (version 6.9, Varian, USA) converted to netCDF format using Vx capture version 2.1 (Adron Systems, Laporte, MN, USA). UPLC-Q-TOF-MS raw data operated by MassLynx software (version 4.1; Waters) were converted into netCDF (*.cdf) format with the Waters DataBridge version 2.1 software. After conversion, the MS data were processed using the Metalign software package (http://www.metalign.nl). This process includes baseline correction, peak detection, peak matching, and retention time alignment. The resulting data were exported to Excel (Microsoft, Redmond, WA, USA), and a statistical analysis was performed.

After the multivariate statistical analysis, major metabolites were positively identified using standard compounds by comparing both the mass spectra and retention time. In LC-MS analysis, the accurate masses and elemental compositions were calculated using the MassLynx software (Waters Corp.) incorporated in UPLC-Q-TOF-MS, MS^n^ fragmentation patterns and UV spectrum were also supported to identify major metabolites using MS workstation software (version 6.9, Varian, USA) in LC-IT-MS/MS. When standard compounds were not available, a tentative identification was performed based on the MS spectra using the NIST05 MS Library (NIST, 2005), combined chemical dictionary version 7.2 (Chapman & Hall/CRC), references, and an in-house library [33], [34]. The metabolomics standards initiative (MSI) has been categorized into four different levels according to [35]. MSI level 1 designates that compounds are compared relatively to an authentic compound analyzed under identical experimental conditions. The putatively annotated compounds based on database information or literature values have been defined as MSI level 2. Unknown analytes have been defined as MSI level 3 (only accurate down to the compound class) and the last is level 4 (unknown, but quantifiable, compounds). Metabolites were quantified by their m/z peak areas calculated using the software provided with the instrument and data processing. This quantification gave relative comparisons of the metabolites according to different ripening of KB.

### Multivariate data analysis and visualization

After data processing, multivariate data analysis was performed using SIMCA-P+ software version 12.0 (Umetrics, Umea, Sweden). Unsupervised PCA and supervised PLS-DA were used to compare fruit ripening of KB samples and to identify the major metabolites related to ripening. Variables were mean centered and unit variance scaled in a column wise manner. Metabolites with a variable importance in the projection (VIP) value>0.7 and a *p*-value<0.05 were selected as suitable metabolites. Significantly different metabolites were represented by box-whisker plots using STATISTICA, version 7.0 (StatSoft Inc., Tulsa, OK, USA). To visualize metabolite profile, heatmap was generated using MultiExperiment Viewer software version 4.8 (http://www.tm4.org/).

### Quantitative real-time PCR

For expression analysis by qRT-PCR, RNA was isolated from three different ripening stage samples and used to generate cDNA. Real-time PCR was performed on ECO™ Real-time PCR system (Illumina, USA) with the SsoFast™ EvaGreen supermix (Bio-Rad, USA) according to conditions described previously [29]. The expression levels of different genes were normalized to the constitutive expression level of ubiquitin. Primer-sequences are listed in Table S1.

### *Arabidopsis* complementation analysis

The *Arabidopsis tt5-1* mutant (CS86, the ABRC) defective in gene encoding CHI was used for transgenic complementation analysis. For generation of overexpressing *RcMCHI2* construct, total RNA isolated from stage 2 fruit was reverse-transcribed using QuantiTect Reverse Transcription Kit (Qiagen, USA). Using this cDNA as template, full-length coding sequence of *R. coreanus CHI2* was amplified by PCR with primer BamHI-CHI2-F (GGATCCTAATGGCTCCACCAATCACC) and AvrII-CHI2-Rev (CCTAfGGTCATGCCTCAACTTCTGCTT). The PCR product, with the 5′ *BamHI* site and 3′ *AvrII* site, was cloned into the pCR-blunt vector (Invitrogen, USA). After digestion with *BamHI* and *AvrII*, the fragment was then cloned into a binary vector, myc-pBA, under the transcriptional control of the CaMV *35S* promoter. The resulting 35Spro:MYC-RcMCHI2 construct was transformed into *Agrobacterium tumefaciens* strains GV3101 and used for stable transformation of *Arabidopsis tt5-1* mutant by floral dip method [36]. *Arabidopsis* tranformants were identified by Basta selection, and lines with a high level of MYC-RcMCHI2 protein were selected by Immunoblot analysis. To induce anthocyanin accumulation, seeds from four independent lines with strong transgene expression were germinated on MS medium without nitrogen sources (PhytoTechnology Laboratories, USA). To confirm the changes of *Arabidopsis* tranformants, anthocyanin analysis was also performed by UPLC-Q-TOF-MS under the same conditions, as used for metabolite profiling of KB in different ripening stages.

### Immunoblot analysis

Total protein was extracted from 100 mg of fresh leaf tissue obtained from 3-week-old plants with 300 ml extraction buffer (50 mM Tris-HCl, pH 8.0, 150 mM NaCl, 5 mM EDTA, 0.2% Triton X-100) containing protease inhibitor cocktail (Roche, USA). Immunoblot analysis was performed as described previously [37]. 20 µg of total protein from each sample was separated on a 12% SDS-PAGE and transferred to PVDF membrane (Millipore, USA). Membranes were blocked with 1×TBST buffer containing 5% -dried milk and then incubated with 1∶2000 diluted primary antibody (mouse anti-c-Myc, GenScript Inc., USA). After three washings with 1×TBST buffer, the blot was incubated with 1∶5000 diluted horseradish peroxidase–conjugated goat anti-mouse antibody (Santa Cruz Biotechnology, USA). The reaction was detected using the ECL detection reagents (Amersham Biosciences, UK).

## Results

### Transcriptome sequencing and *de-novo* assembly

**Figure 1:**
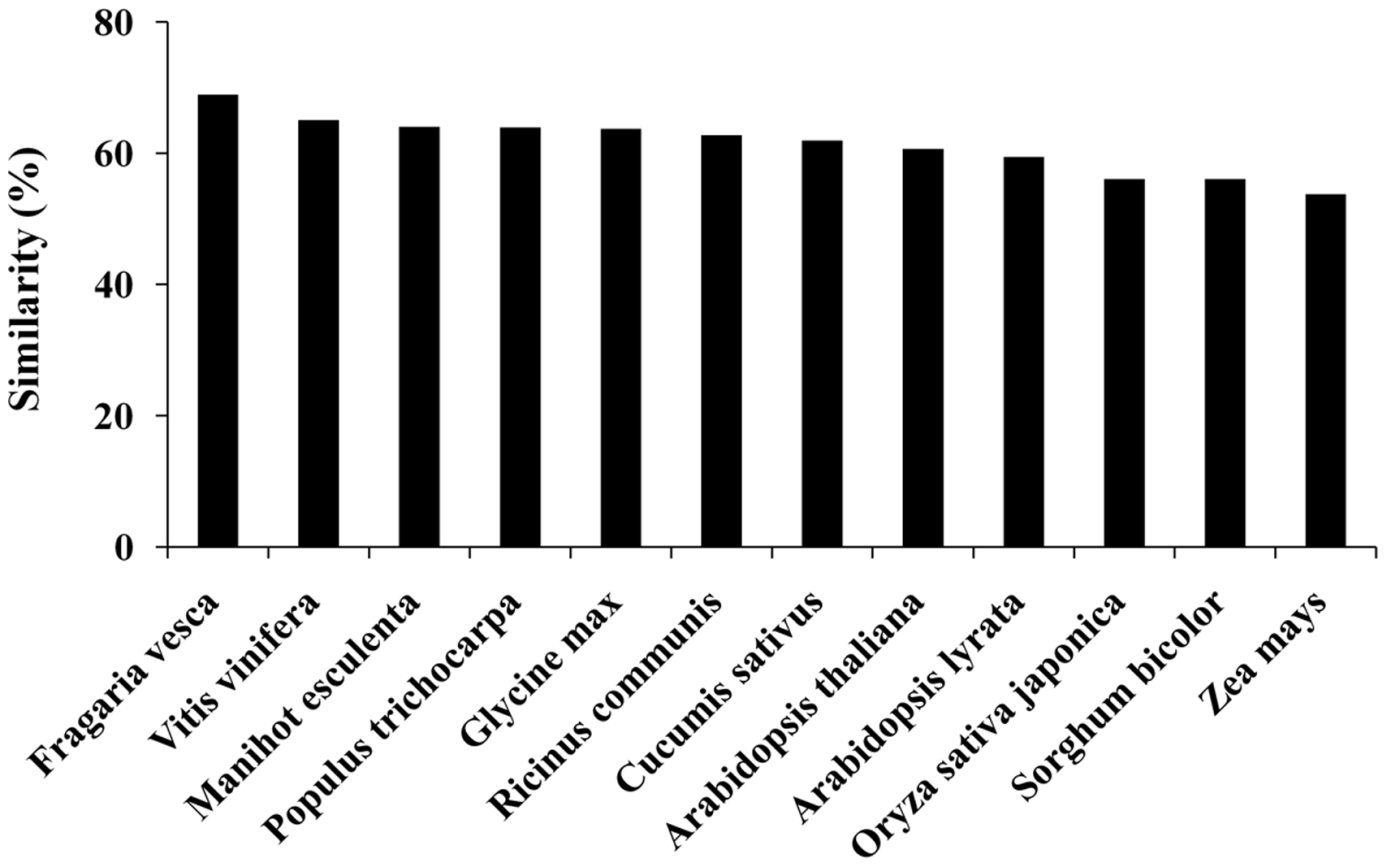
The relative sequence conservation of Korean black raspberry transcripts with other plant species. The KB transcripts (coverage≥70%) were searched against proteome sequences using BLASTX program for analyzing sequence similarity. The percentage of transcripts showing significant similarity (E-value<10^−5^) in BLASTx search are shown in represented by bar diagram.

Normalized cDNA library prepared from the total RNA of *R. coreanus* Miquel fruit (20 DAF) was subjected to pair-end read with the Illumina platform. After removal of adaptor sequences, ambiguous reads and low-quality reads (Q20<20), 54.08 million reads comprised total of 4,867,515,000 nucleotides (>4.86 Gb) were obtained for assembly (Table 1). Trinity program [32] was used to assemble all high-quality reads into a total of 44,619 contigs with an average length of 755 bp, with half of the total assembly length in contigs >1.1 kb (N50 = 1,155 bp). 23,393 contigs (46.57% of contigs) were at least 500 bp in length, and 22.97% of contigs were longer than 1,000 bp (Figure S2A). The contigs were then joined into 43,723 unigenes, with an average length of 754 bp and an N50 of 1,153 bp, using TIGR Gene Indices Clustering tool (Table 1). The unigenes distribution closely followed the contigs distribution (Figure S2B). The sequence conservation and similarity analysis is quite useful for transferring knowledge from model plants to non-model plants [38]. The transcript set of KB was analyzed for similarity against the proteome data sets for 12 plants including *Arabidopsis*, rice, cucumber and grape using BLASTX search (E-value cut-off threshold of ≤1E-05). As shown in Figure 1, KB transcriptome showed 68.9% similarity with strawberry (*Fragaria versa*) followed by 65% with grape vine (*Vitis vinifera*). As predicted, lesser number of KB transcripts exhibited similarity with monocots proteomes (53.7-56%) as compared to dicots (59.4–68.9%). The phylogenetic tree analysis of the known *Rosaceae* family members based on DNA sequence data of nuclear and chloroplast genomic regions has suggested that *Fragaria* and *Rubus* are members of the sub-family *Rosoideae*, which have the same basal chromosome number of x = 7 [1], [39]. Thus, the significant homology between strawberry proteins and KB transcriptome suggests evolutionary relationship between them, and they also might have conserved functions.

**Table 1:** Overview of the sequencing and assembly.

	Korean black raspberry
Total number of reads	54,083,500
Total nucleotides (nt)	4,867,515,000
GC percentage	47.61
Q20 percentage	96.11
Step-wise assembly	
Trinity	
Total number of trinity	44,619
Length of all trinity (nt)	33,708,690
Average sequence size of trinity (nt)	755
Trinity N50 (nt)	1,155
Unigene	
Total number of unigenes	43,723
Length of all unigenes (nt)	32,956,464
Average sequence size of unigenes (nt)	754
Unigenes N50 (nt)	1,153

### Functional annotation of Korean black raspberry transcriptome

For annotation of assembled unigenes, distinct gene sequences were searched using BLASTX against NCBI non-redundant (NR) database with a cut-off E-value of 10^−5^. Out of all 43,723 KB unigenes, 29,955 (68.51% of all unigenes) had BLAST hits to known proteins in NR database (Tables 2 and S2). In addition, all unigenes were aligned to public protein database including Swiss-Prot, Kyoto Encyclopedia of Genes and Genomes (KEGG), Cluster of Orthologous Groups (COG) and Gene ontology (GO) by BLASTX (cut-off E-value of 10^−5^). As summarized in Table 2, total 30,178 unigenes (69.02%) were annotated in this manner, whereas the rest could not be matched to known genes due to the sequence contamination and limitation of genome and EST information in *Rubus*. Human, bacterial and viral sequences contamination was investigated using the web-based version of DeconSeq (http://deconseq.sourceforge.net/) [40], with a query coverage and sequence identity threshold of 90%. Among the KB sequencing reads, we found low contamination (about 1.11% of total reads), indicating that most of unigenes, which did not matched to known genes, might be specific genes for *Rubus* genus and *Rosaceae* family.

**Table 2:** Summary of annotations used for the Korean black raspberry unigenes in public protein databases.

Public protein database	No. of unigene hit	Percentage
NR	29,955	68.51
KEGG	15,977	36.54
COG	10,480	23.97
Swiss-Prot	21,964	50.23
GO	11,253	25.74
Total	30,178	69.02

In case of gene functional classification, a total of 11,253 (25.74%) unigenes were assigned with at least one GO term. Interestingly, 18,989 unigenes were assigned with at least one GO term in biological category, 21,786 unigenes in cellular component category and 11,193 unigenes in molecular function category (Figure S3). To further evaluate the completeness of KB transcriptome library, the assembled unigenes were searched against COG. Out of 29,955 NR hits, 10,480 of the assembled sequences were assigned to the COG classification (Table 2). Among 25 functional categories, the five major categories with maximum unigenes coverage are as follows; “General function prediction only” (3,437 unigenes) associated with basic physiological and metabolic functions, “Transcription” (2,013 unigenes), “Replication, recombination and repair” (1,780 unigenes), “Posttranslational modification, protein turnover, chaperones” (1,525 unigenes) and “Signal transduction mechanisms” (1,381 unigenes), while only few unigenes were assigned to “Extracellular structures” and “ Nuclear structure” (Figure S4). Furthermore, 534 unigenes were classified into the group of “Secondary metabolites biosynthesis, transport and catabolism”. Taken together, these results suggest that the *de-novo* assembled unigenes of KB have wide transcriptome coverage, and provide a valuable resource for facilitating the discovery of novel genes involved in the specific physiological and developmental processes.

### Metabolite profiling of Korean black raspberry fruits at different ripening stages

**Figure 2:**
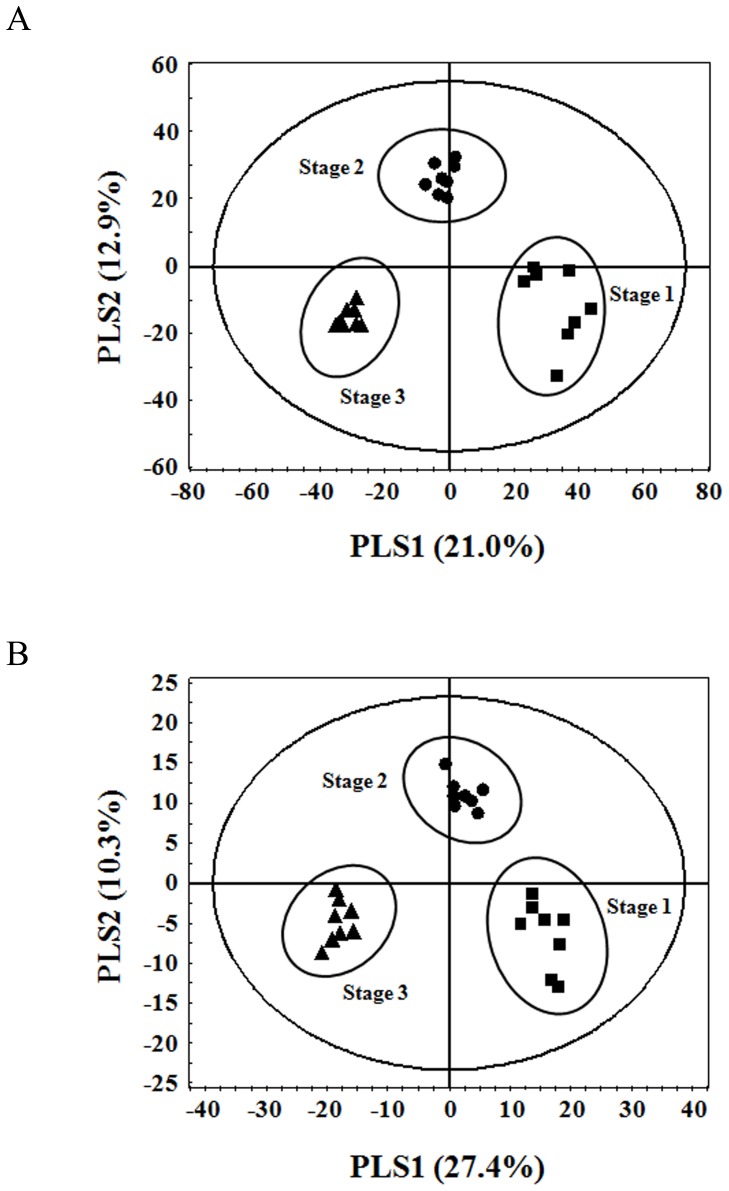
PLS-DA score plots of metabolite profiles in three different ripening stages of Korean black raspberry. The datasets obtained by GC-IT-MS (A) and UPLC-Q-TOF-MS (B) were analyzed by PLS-DA.

**Figure 3:**
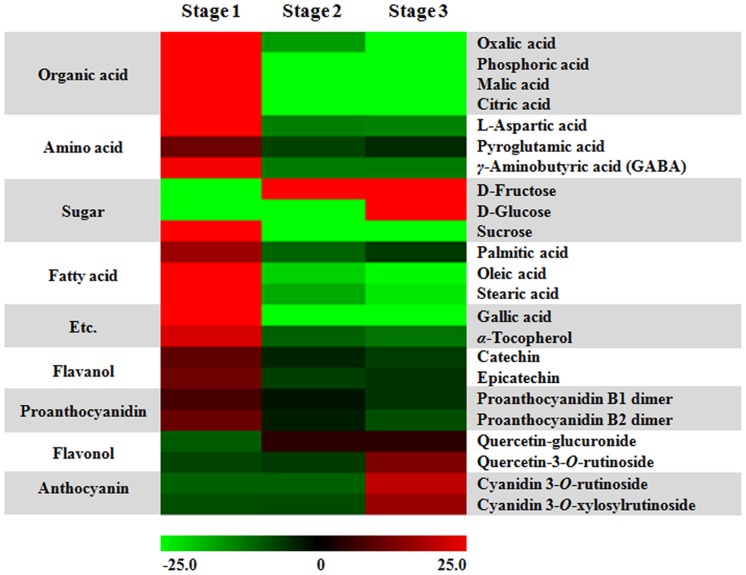
The key metabolites identified from PLS-DA in different ripening stages of Korean black raspberry. 23 metabolites that show statistically significant changes during ripening process (VIP>0.7 and *p*<0.05) were represented by Heatmap, with relative intensities indicated by the heat scale.

Mass spectrometry based metabolite profiling of KB at three ripening stages, 15 days post-anthesis (DPA) (stage1, commonly used in traditional herbal medicine), 20 DPA (stage 2) and 25 DPA (stage 3, used as fresh fruit and for processing), was performed to investigate the changes in metabolic composition (Table S3 and S4). To support pattern recognition of the metabolic differences in KB samples according to the different ripening stages, the data sets were statistically analyzed by principal component analysis (PCA) and partial least squares discriminant analysis (PLS-DA). As shown in Figure 2, PLS-DA score plots from both data sets point toward differential metabolite profile between samples harvested at different stages. Similar patterns were shown in the PCA (Figure S5). In GC-IT-MS analysis, stage 1 (positive PLS1 dimension) fruits were clearly separated from stage 3 (negative PLS1 dimension) along PLS1 (21.0%), whereas stage 2 fruits were separated from stage 1 and 3 along PLS2 (12.9%), (Figure 2A). A total of fifteen metabolites were selected as differential variables using VIP (variable importance in the projection) value (VIP>0.7) and *p* value (*p*<0.05) from PLS-DA dataset (Table 3). Primary metabolites, including organic acids, sugars, fatty acids and amino acids, were found to influence cluster formation of GC-IT-MS profiles. The heatmap represents the differential distribution of metabolites during ripening of fruit (Figure 3). In comparison to fruit expansion, the progress of fruit ripening has been characterized by a decrease in sucrose, organic acids and GABA [41]. Similarly, our finding demonstrated the decrease in sucrose, most of the amino acids, organic acids and fatty acids, whereas fructose and glucose increased in relative concentration during ripening of fruit, indicating that KB underwent major changes in carbon-nitrogen metabolism during fruit ripening.

**Table 3:** Differential metabolites identified in Korean black raspberry during ripening stages analyzed by GC-IT-MS.

No.	RT (min)^1^	VIP value	Identified ion (*m/z*)^2^	Putative metabolites^3^	Derivatized^4^	*p*-value	ID^5^
1	4.41	1.21	147	oxalic acid	(TMS)_2_	0.005	STD
2	6.08	1.15	299	phosphoric acid	(TMS)_3_	0.003	STD
3	8.91	1.09	147	malic acid	(TMS)_3_	0.021	STD
4	9.32	0.95	147, 232	L-aspartic acid	(TMS)_3_	0.023	STD
5	9.42	1.33	156	pyroglutamic acid	(TMS)_2_	0.005	STD
6	9.46	1.17	174	γ-aminobutyric acid (GABA)	(TMS)_3_	0.002	STD
7	12.66	1.46	273	citric acid	(TMS)_4_	0.005	STD
8	13.16	1.33	217	D-fructose	MeOX (TMS)_5_	0.034	STD
9	13.42	1.70	148, 217	D-glucose	MeOX (TMS)_5_	0.000	STD
10	14.06	1.24	281	gallic acid	(TMS)_4_	0.000	STD
11	15.02	1.07	313	palmitic acid	TMS	0.013	STD
12	16.57	1.49	339	oleic acid	TMS	0.001	STD
13	16.78	1.63	117,341	stearic acid	TMS	0.000	STD
14	19.72	1.16	361	sucrose	(TMS)_8_	0.004	STD
15	23.65	1.44	237	α-tocopherol	TMS	0.000	STD

1 Retention time.

2 m/z are the selected ion(s) for identification and quantification of individual derivatized metabolites.

3 Identified metabolites based on variable importance projection (VIP) analysis with cut-off value of 0.7 and a p-value<0.05.

4 MEOX, methyloxime; TMS, trimethylsilyl.

5 Identification: STD, mass spectrum was consistent with that of standard compound (MSI level 1).

The resulting score plot from PLS-DA of the UPLC-Q-TOF-MS analysis showed that fruits from different ripening stages were clearly distinguished by combining PLS1 (27.4%) and PLS2 (10.3%), (Figure 2B). Eight flavonoid-derived secondary metabolites including flavanols, flavonols, proanthocyanidins and anthocyanins were significant metabolites for relative analysis among three ripening stages of fruits (Table 4). The amount of flavanols (catechin and epicatechin) and B type proanthocyanidin dimers was decreased, whereas flavonols (quercetin 3-*O*-rutinoside and quercetin-glucuronide) and anthocyanins (cyanidin 3-*O*-xylosylrutinoside and cyanidin 3-*O*-rutinoside) contents were increased during the progress of ripening (Figure 3). In particular, it was found that the major anthocyanin in the ripening KB is cyanidin derivatives, which was highly accumulated in dark red fruit (stage 3).

**Table 4:** Differential metabolites identified in Korean black raspberry during ripening stages analyzed by UPLC-Q-TOF-MS and LC-IT-MS/MS.

			UPLC-Q-TOF-MS			LC-IT-MS/MS						
No.	RT^1^	VIP value	Experimental mass [M-H]−	Formula	Δppm	[M-H]^−^	[M+H]*^+^*	MS^n^ fragment ions (*m/z*)	UV(nm)	Tentatively metabolites^2^	*p*-value	ID^3^
1	3.06	1.71	725.1926	C_32_H_37_O_19_	−0.4	725	727	727>287>213	276, 519	cyanidin 3-O-xylosylrutinoside	0.000	DB
2	3.13	1.72	593.1504	C_27_H_29_O_15_	−0.3	593	595	595>287>213	278, 518	cyanidin 3-O-rutinoside	0.000	STD
3	3.16	1.67	577.1336	C_30_H_25_O_12_	−1.7	577	579	577>289>188	248, 314	proanthocyanidin B1 dimer	0.000	DB
4	3.28	1.60	289.0712	C_15_H_13_O_6_	0.0	289	291	291>123	274, 306	catechin	0.000	STD
5	3.38	1.62	577.1348	C_30_H_25_O_12_	0.3	577	579	577>289>188	242, 301	proanthocyanidin B2 dimer	0.000	DB
6	3.57	1.32	289.0711	C_15_H_14_O_6_	−0.3	289	291	291>123	272, 300	epicatechin	0.000	STD
7	3.90	1.65	609.1461	C_27_H_29_O_16_	0.8	609	611	609>301>151	275, 351	quercetin 3-O-rutinoside	0.000	STD
8	4.18	0.95	477.0679	C_21_H_17_O_13_	2.1	477	479	477>301>179	269,358	quercetin-glucuronide	0.002	DB

1 Retention time.

2 Identified metabolites based on variable importance projection (VIP) analysis with cutoff value of 0.7 and a *p*-value<0.05.

3 Identification: STD, standard compound (MSI level 1); DB, database (MSI level 2).

### Identification of candidate enzymes involved in anthocyanin biosynthesis

**Figure 4:**
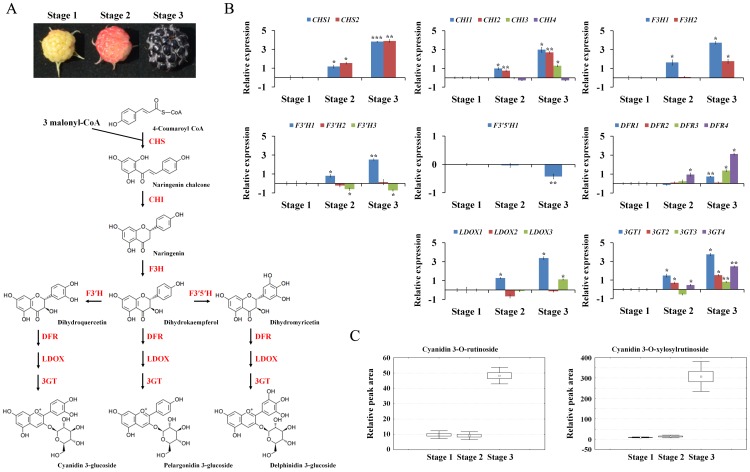
The relative gene expression and subsequent anthocyanin amounts produced during the ripening process. (A) Biosynthetic pathway of anthocyanins. Enzyme names were abbreviated as follows: chalcone synthase (CHS), chalcone isomerase (CHI), flavanone 3-hydroxylase (F3H), flavonoid 3′-hydroxilase (F3′H), flavonoid 3′5′-hydroxylase (F3′5′H), dihydroflavonol 4-reductase (DFR), leucoanthocyanidin dioxygenase (LDOX) and anthocyanidin 3-O-glucosyltransferase (3GT). (B) Expression pattern of genes involved in anthocyanin biosynthesis pathway. Expression levels of genes from ripening stage 2 and 3 were compared to stage 1. The means and standard errors were calculated from three independent measurements. Student's t test compared with Stage 1, * P<0.05, ** P<0.01 and *** P<0.001. (C) Anthocyanin contents determined by UPLC-Q-TOF-MS were represented by box-whisker plots. Metabolites were relatively quantified by their m/z peak areas with the instrument and data processing. The error bars are the standard deviations from three independent measurements.

The accumulation of cyanidin derivatives during ripening process prompted us to investigate whether this phenomenon is mediated by altering expression of flavonoid metabolic pathway genes. The detailed anthocyanin biosynthesis pathway has been illustrated in Figure 4A [42]. The anthocyanin biosynthetic pathway is usually divided into two sections, the early and later sections. As per our prediction, based on the KEGG pathway assignment, we found 28 unigenes encoding 23 putative enzymes involved in anthocyanin biosynthesis from KB transcriptome library (Table 5). Although most of them were having partial nucleotide sequences, we were able to analyze their expression pattern during ripening process using qRT-PCR. As illustrated in Figure 4B, the expression level of most unigenes such as chalcone synthases (CHSs), chalcone flavanone isomerases (CHIs), and flavanone 3-hydroxylases (F3Hs) regulating dihydrokaempferol biosynthesis increased significantly when the fruits turned dark red (stage 3). Similarly, the enhanced transcript level of *DFRs* (dihydroflavonol 4-reductases), *LDOXs* (leucoanthocyanidin dioxygenases) and *3GTs* (anthocyanidin 3-O-glucosyltransferases) unigenes was also noticed, which leads to the formation of anthocyanins. In addition, the expression of *F3′H1* (flavonoid 3′-hydroxylase1) showed a dramatic increase during ripening process. On the other hand, *F3′5′H* (flavonoid 3′5′-hydroxylase) unigene was down-regulated from 15 DPA to 25 DPA. This is consistent with the accumulation of cyanidin derivatives but not delphinidin derivatives (Figure 4C). One speculation is that the increased level of *F3′H1* might have accumulated cyanidin derivatives as major anthocyanin compounds due to the enzymatic competition for dihydrokaempferol substrate.

**Table 5:** Potential unigenes related to anthocyanin biosynthesis pathway.

			*Rubus coreanus*			
Gene	EC number	Gene Name^1^	NU	MNPU	NGSN	*Fragaria vesca* 2
**Chalcone synthase**	EC:2.3.1.74	CHS	2	2	-	FV7G02590
						FV7G02600
**Chalcone isomerase**	EC:5.5.1.6	CHI	4	4	-	FV7G31110
						FV7G25290
						FV2G25040
						FV3G19830
**Flavanone 3-hydroxylase**	EC:1.14.11.9	F3H	2	2	EU078685	FV1G13680
					EU255776	FV6G51040
**Dihydroflavonol-4-reductase**	EC:1.1.1.219	DFR	5	4	-	FV2G34030
						FV7G11200
						FV3G23500
						FV2G50610
**Flavonoid 3′-hydroxylase**	EC:1.14.13.21	F3′H	5	3	-	FV5G12250
						FV1G10430
						FV0G15960
**Flavonoid 3′,5′-hydroxylase**	EC:1.14.13.88	F3′5′H	1	1	-	FV5G00680
**Leucoanthocyanidin dioxygenase**	EC:1.14.11.19	LDOX	3	3	-	FV5G01390
						FV5G18660
						FV7G29560
**Anthocyanidin 3-O-glucosyltransferase**	EC:2.4.1.115	3GT	6	4	-	FV7G33970
						FV7G06650
						FV5G37930
						FV3G11420
**Total**			28	23	2	

1 Following the KEGG nomenclature.

2 Homologous genes from *Fragaria vesca*.

**NU**, number of unigenes; **MNPU**, maximum number of proteins encoded by unigenes; **NGSN**, number of genes stored in NCBI.

To generate a flux map of anthocyanin biosynthesis during the ripening process, the analysis of metabolic flux was carried out using YANA tool [43]. The shift in anthocyanin biosynthesis resulted from the differential gene expressions during the ripening process has been shown in Figure S6, which suggests that DFR4 and LDOX1 are major enzymes for the biosynthesis of cyanidin derivatives.

### Functional characterization of chalcone isomerase in *R. coreanus* Miquel transcriptome

**Figure 5:**
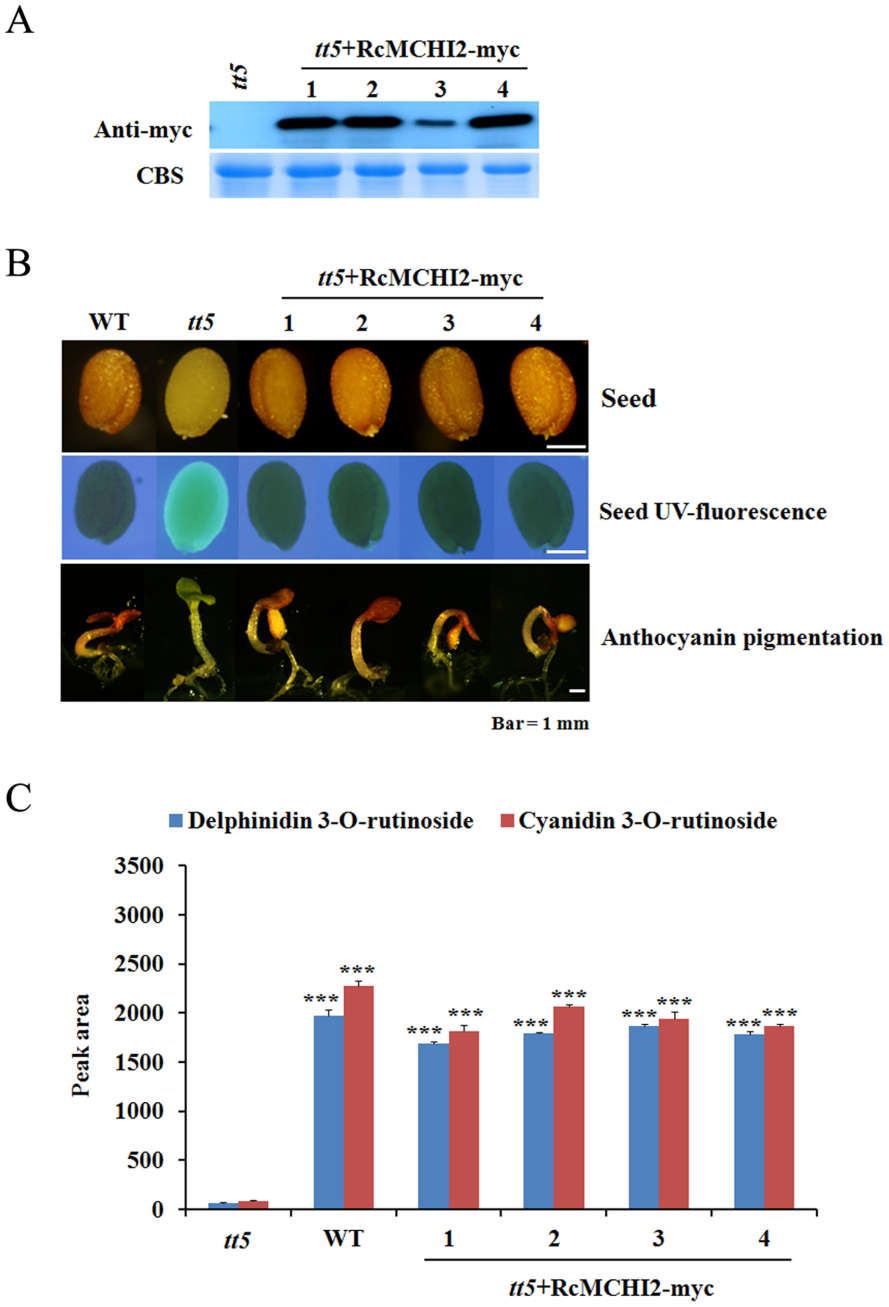
Korean black raspberry chalcone isomerase 2 (*RcMCHI2*) rescues *Arabidopsis tt5* mutant. (A) The expression of MYC-RcMCHI2 was confirmed by western blotting with anti-myc tag antibody in two weeks old T1 plants. (B) Restoration of anthocyanin pigmentation and seed coat color in the transgenic *Arabidopsis tt5-1* mutants expressing *RcMCHI2*. Seedlings were grown in low nitrogen medium to induced anthocyanin accumulation. The mature dried seeds were exposed to UV. (C) The analysis of anthocyanins in extracts of RcMCHI2 transgenic *Arabidopsis tt5-1* lines. The relative area of anthocyanins in 1-week-old T2 seedlings was calculated by MassLynx software. The error bars are the standard deviations from three independent measurements. Student's t test compared with *tt5* mutant, *** P<0.001.

The distribution of CHI in higher plants results in phenotypic color changes [44]–[46]. In addition, it has been suggested that a major limitation in the flavonoid biosynthetic pathway in tomato fruit is the lack of *CHI* expression [47], [48], indicating the significance of this enzyme for the flux of the flavonoid pathway including anthocyanin biosynthesis. From KB transcriptome library, we found a full-length CHI unigene (*RcMCHI2*, Unigene18325), together with three fragment sequences encoding different CHI proteins. CHIs are mainly classified into two types, type I CHI and type II CHI [49]. Type I CHIs isomerizes only naringenin chalcone to produce naringenin, whereas type II CHIs can catalyze both naringenin chalcone and isoliquiritigenin into naringenin and liquiritigenin, respectively [49]. A phylogenetic tree generated by the neighbor-joining method based on the amino acid sequences exhibited that RcMCHI2 is evolutionally related to type I CHI group (Figure S7). The *Arabidopsis transparent testa 5-1* (*tt5-1*) mutant, lacking CHI activity, was selected to functionally characterize the RcMCHI2. *RcMCHI2* carrying Myc-tag was placed under the control of the 35S promoter and transformed into the *Arabidopsis tt5-1* mutant. Transgene expression was confirmed by western blotting with anti-myc tag antibody, and four independent lines were selected for phenotype investigations (Figure 5A). The seeds collected from selected transgenic lines showed pigmentation characteristic of the wild-type *Arabidopsis*, while seeds of the *Arabidopsis tt5-1* mutant were yellow in color (Figure 5B). Dried seeds of *Arabidopsis tt5-1* mutant on excitation with UV-A exhibited a greenish-blue fluorescence that was rescued with the expression of *RcMCHI2*. In addition, these transgenic plants showed anthocyanin pigments in the cotyledon and hypocotyls when grown under nitrogen-deficient condition, unlike *Arabidopsis tt5-1* seedlings (Figure 5B). Also, the ectopic expression of *RcMCHI2* in *Arabidopsis tt5-1* lines accumulate similar amount of delphinidin 3-*O*-rutinoside and cyanidin 3-*O*-rutinoside, as compared to wild-type *Arabidopsis* seedlings (Figure 5C; Table S5). These findings strongly suggest that *RcMCHI2* encodes functional CHI enzyme, which strongly support that our KB transcriptome library provides a solid foundation for further functional and genomics research.

## Discussion

The comprehensive overview of metabolome based upon the existence of actual transcripts is the powerful approach for identifying the actual biochemical status of samples. In addition, the identification of interrelations between metabolites and genes or proteins can facilitate the understanding of molecular mechanisms involved in various physiological and developmental processes.

In this study, to accomplish comparative analyses during fruit ripening process of *R. coreanus* Miquel, we have exploited both transcriptomic and metabolomic approaches. Here, we firstly used RNA-seq technology to generate the transcriptome library on the Illumina HiSeq™2000 sequencing platform. Based on *de-novo* sequencing and assembly, we obtained 54.08 million high-quality reads, and identified a total of 43,723 unigenes (Table 1). Among them, 30,178 unigenes (about 69% of the assembled unigenes) were annotated in one or more of the public databases (Table 2). To the best of our knowledge, this is the first attempt using Illumina sequencing platform for *de-novo* sequencing and assembly of KB without a reference genome. *Fragaria* and *Rubus* have similar fruit form and show close relation of nuclear DNA phylogenies [39] and morphology [50]. In addition, we also found the significant homology between strawberry and KB transcriptome sequences (Figure 1), suggesting the colinearity between the *Fragaria* and *Rubus* genomes. Comparison of assembled genes to gene catalogs, functional annotation and classification (Figures S3 and S4) indicates that the transcriptome library generated from KB can provide a very efficient means for identification of functional genes, and facilitate the investigation of interrelations between metabolites and genes.

Traditionally, unripe KB has been used as a resource of herbal medicine for treatment of spermatorrhea and allergic diseases, while ripe fruits are generally used as the economically important fresh and processed fruits in East Asia [11], [51]. This indicates that metabolites of KB vary with the fruit maturation process. Fruit ripening is a highly complex process that involves the coordinated regulation of various metabolic pathways including hormonal regulation [52], pigmentation [13], sugar metabolism [41] and cell wall metabolism [15]. The decreased sucrose and increased glucose and fructose levels have been observed during the ripening process of various plants [10], [41], [53], [54]. Similarly, KB also exhibited the major shifts in primary metabolites during ripening process (Figure 3). The alteration of enzymes activities including sucrose synthase, fructokinase, glucokinase and invertase, which are required to mobilize sucrose and reducing sugars, regulates the progressive increase of hexoses and decline of sucrose amount during fruit development and ripening [55], [56]. Therefore, the increasing level of hexoses indicates that the major shifts in primary metabolites mediated by enzymes activity may be a common feature of fruit ripening. In addition, the resulting score plot from PLS-DA of the GC-IT-MS suggested that organic acids such as citric acid and malic acid have the high impact on the metabolic shift (Table 3; Figure 3). Citric acid and malic acid, which are tricarboxylic acid (TCA) cycle intermediates, are highly correlated to many important regulators of fruit ripening [14], [57]. Citric acid produced by the TCA cycle can be degraded in the cytosol through GABA synthesis pathway, which is also known as a GABA shunt [58]. In tomato, GABA is converted to malic acid via succinate semialdehyde, passes into a shunt comprised of oxaloacetate-phosphoenolpyruvate-pyruvate and TCA cycle, and then is stored as citric acid [59]. The alternating contents of citric acid, malic acid and GABA during ripening process of various fruits including KB may reflect a change in the anapleurotic capacity of the cycle [57], [60].

Anthocyanins have been shown to be the predominant phenolic compounds, which have strong antioxidant capacity, in the fruits of berries, including blackberries, blueberries, strawberries and grapes [61], [62]. During the progression of ripening, many berry species accumulate anthocyanin in their fruits, whereas the amounts of monomeric (−)-epicatechin and (+)-catechin, dimeric, oligo- and polymeric proanthocyanidins have been found to be decreased [13], [60], [63]. In KB, same phenomenon has been observed in fruit samples at different ripening stages (Figure 3), indicating that anthocyanins are potent marker for ripening and organoleptic quality. Furthermore, the switch in flavonoid biosynthesis from proanthocyanidins to anthocyanins during ripening process of KB might reflect the shift in strategy from protecting fruit to promoting fruit consumption by herbivory in later stage for seed dispersal. The anthocyanin biosynthesis pathway has been extensively studied in several plant species, including petunia (*Petunia hybrid*), pears (*Pyrus communis* L.), and bilberry (*Vaccinium myrtillus*) [48], [63], [64]. In our *R. coreanus* transcriptome database, we identified 28 unigens encoding 23 putative enzymes involved in anthocyanin biosynthesis pathway (Table 5). Anthocyanin accumulation was positively associated with the expression of anthocyanin biosynthetic genes in KB (Figure 4). Although *F3′H1* was significantly up-regulated during ripening process, it might be sufficient for the accumulation of cyanidin derivative as a dominant anthocyanin in KB. As CHI catalyzes a key step for the flux in the flavonoid biosynthesis pathway, it has been suggested as the potential candidate gene for the modification of fruit nutrition and pigmentation through biotechnological applications and metabolic engineering [47], [48]. Using our *R. coreanus* transcriptome database, we found a full-length *CHI* unigene (*RcMCHI2*, Unigene18325), which belongs to type I CHI group (Figure 5). *Arabidopsis* CHS, CHI and DFR were shown to be involved in orientation-dependent interaction, suggesting that enzymes in flavonoid biosynthesis pathway assemble as a macromolecular complex [65]. *RcMCHI2* gene has complemented *Arabidopsis tt5* mutant, restoring this mutant to accumulate anthocyanin in seed coats and seedlings with similar amounts as found in wild type *Arabidopsis*. (Figure 5B and 5C), indicating that CHI can be functionally exchangeable between plants separated by large evolutionary distances [66].

In conclusion, by combining metabolomic and transcriptomic approaches, we have monitored the alteration of several major groups of metabolites during ripening process of KB. Based on *de-novo* transcriptome analysis and functional annotation of novel genes, we have identified transcripts that encode most of the known enzymes involved in anthocyanin biosynthesis in KB, and suggested the positive correlation between the expression of anthocyanin biosynthetic genes and anthocyanin accumulation during ripening process. Our application of RNA-seq to understand gene-to-metabolite networks in fruit ripening process demonstrates the utility of this transcriptome resource. These resources, together with metabolic profiling, would be of great importance for further studies to identify target genes underlying agronomic traits and nutritional quality.

## Supporting Information

Figure S1 **Agilent bioanalyzer gel-like image of total RNA.** The image shows the total RNA gel like-image produced by the Bioanalyzer.
(TIF)

Figure S2 **Length distribution of assembled contigs (A) and unigenes (B).**
(TIF)

Figure S3 **Gene ontology classification of assembled unigenes.** The results are summarized in three main categories: Biological process, Cellular component and Molecular function.
(TIF)

Figure S4 **Histogram presentation of clusters of orthologous groups (COG) classification.** All unigenes were aligned to COG database to predict and classify possible functions.
(TIF)

Figure S5 **PCA score plots of metabolite profiles in three different ripening stages of Korean black raspberry.** The datasets obtained by GC-IT-MS (A) and UPLC-Q-TOF-MS (B) were analyzed by PCA.
(TIF)

Figure S6 **The flux map of anthocyanin biosynthesis during the ripening process.** The metabolic flux was analyzed using YANA tool. The numbers along with protein indicate the predicted enzymatic activity of each protein, which was generated by YANA tool.
(TIF)

Figure S7 **A phylogenetic tree of RcMCHI2 and CHIs from other plant species homology.** Amino acid sequences were analyzed using CLUSTALW alignment in PHYLIP format clustal algorithm. Bootstrap values were presented as a percent of 100 resampled trees at each tree node using default settings of the TreeTop-Phylogenetic Tree Prediction (http://www.genebee.msu.su/services/phtreereduced.html). GenBank accession numbers of CHIs from different plant species: *Arabidopsis thaliana* (At3g55120), *Vitis vinifera* (CAA53577), *Oryza sativa* (AF474922), *Citrus sinensis* (BAA36552), *Medicago sativa* (P28012), *Phaseolus vulgaris* (P14298), *Lotus japonicas* (BAC53983), *Pueraria lobata* (Q43056).
(TIF)

Table S1 **Primer sequences for qRT-PCR analysis.**
(DOC)

Table S2 **Top BLAST hits from NCBI nr database.** BLAST results against the NCBI nr database for all the distinct sequences with a cutoff E-value above 10^−5^ are shown.
(XLS)

Table S3 **MS data from GC-IT-MS analysis after data processing by Metalign software.**
(XLS)

Table S4 **MS data from UPLC-Q-TOF-MS analysis after data processing by Metalign software.**
(XLS)

Table S5 **Identification of anthocyanins in Korean black raspberry** ***CHI2*** **rescued** ***Arabidopsis tt5*** **mutant.**
(DOC)
