# Supplementary figures and images for: De-novo RNA Sequencing and Metabolite Profiling to Identify Genes Involved in Anthocyanin Biosynthesis in Korean Black Raspberry (Rubus coreanus Miquel)
Source: PLoS One. 2014 Feb 5;9(2):e88292. doi: 10.1371/journal.pone.0088292 (PMC3914977; doi:10.1371/journal.pone.0088292)

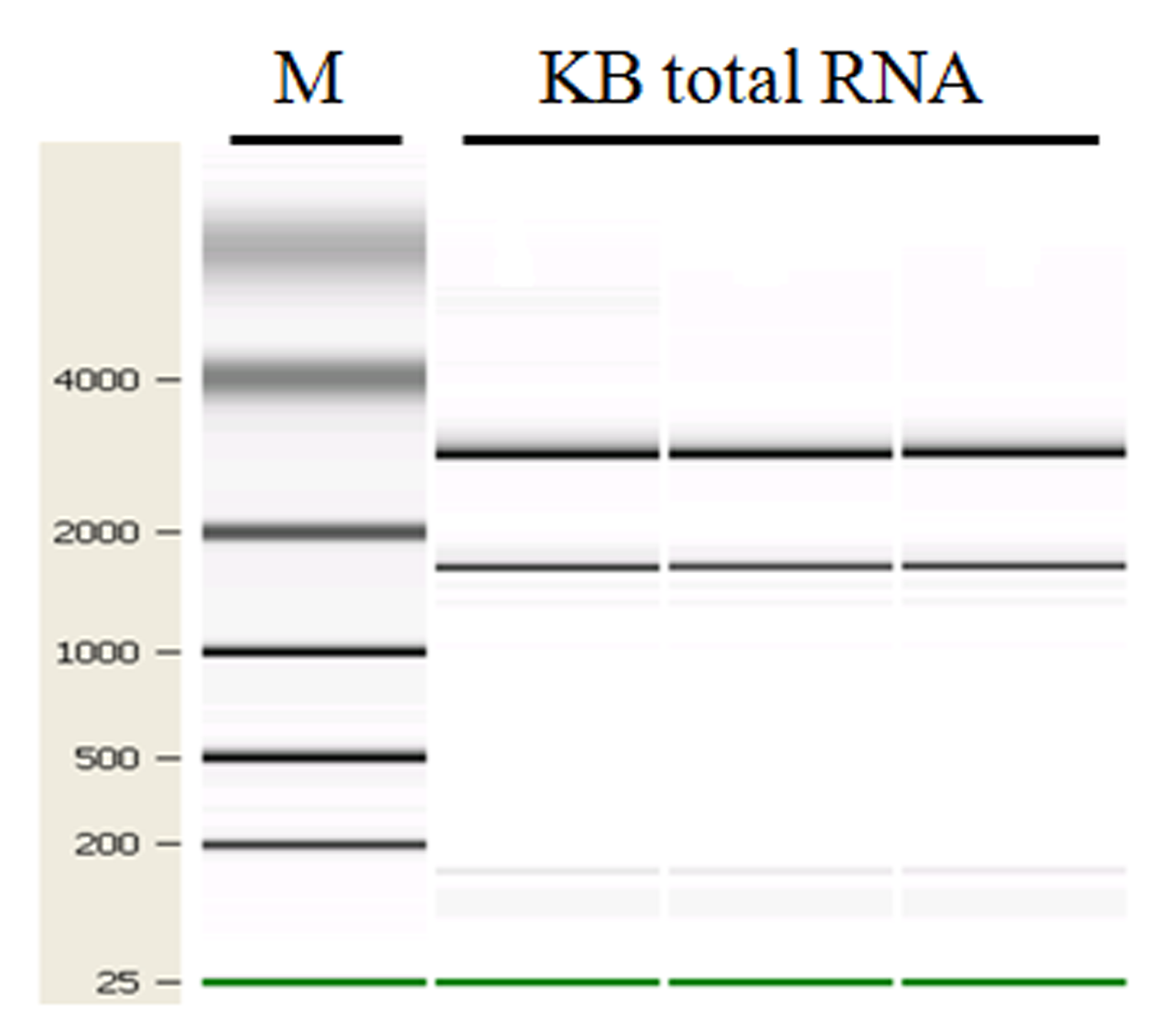

Supplement: Figure S1 — Agilent bioanalyzer gel-like image of total RNA. The image shows the total RNA gel like-image produced by the Bioanalyzer. (TIF) [file pone.0088292.s001.tif]

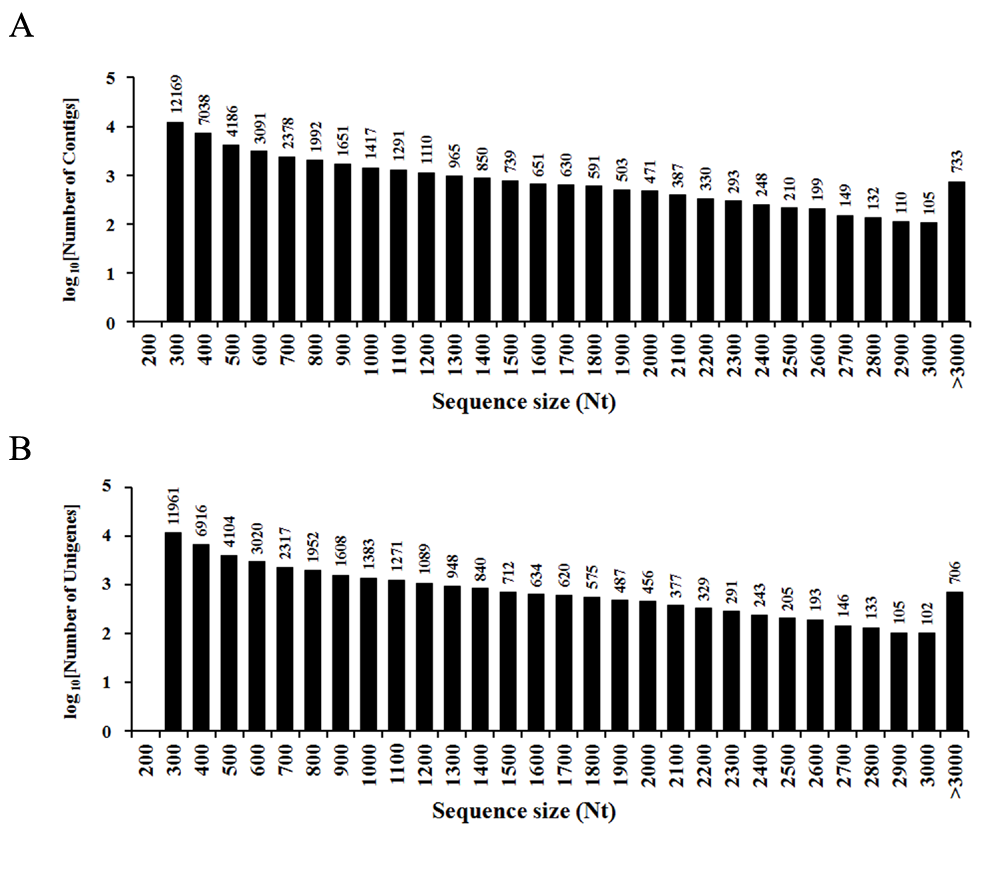

Supplement: Figure S2 — Length distribution of assembled contigs (A) and unigenes (B). (TIF) [file pone.0088292.s002.tif]

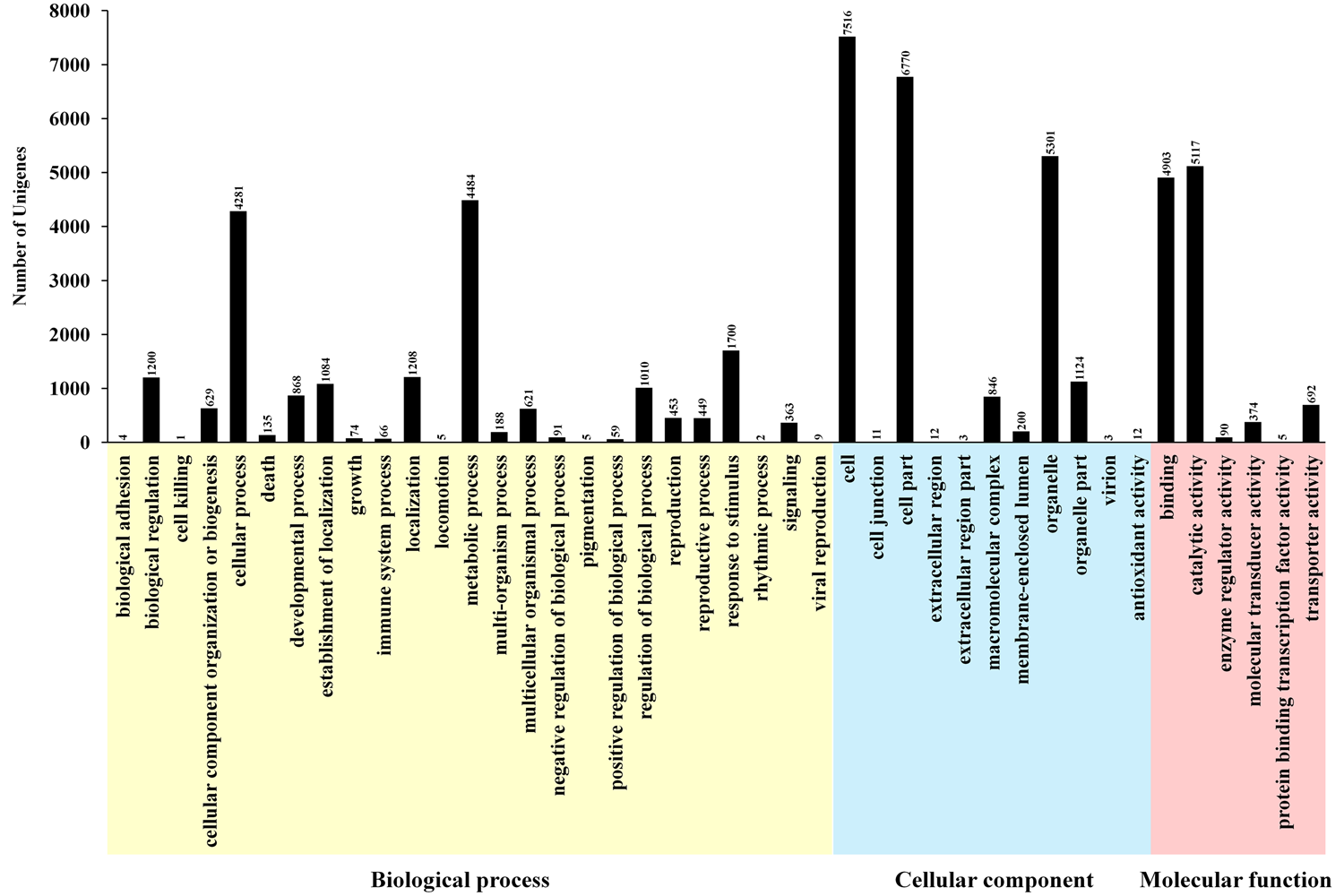

Supplement: Figure S3 — Gene ontology classification of assembled unigenes. The results are summarized in three main categories: Biological process, Cellular component and Molecular function. (TIF) [file pone.0088292.s003.tif]

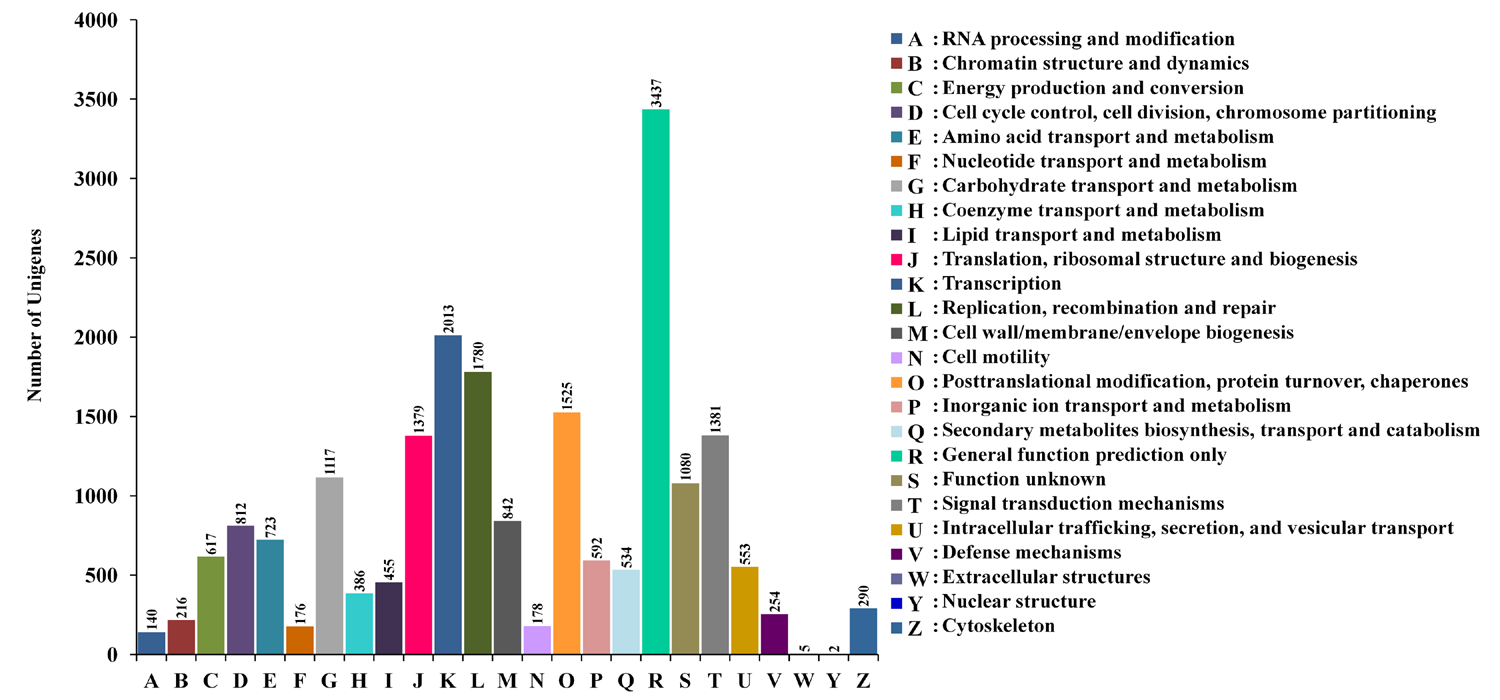

Supplement: Figure S4 — Histogram presentation of clusters of orthologous groups (COG) classification. All unigenes were aligned to COG database to predict and classify possible functions. (TIF) [file pone.0088292.s004.tif]

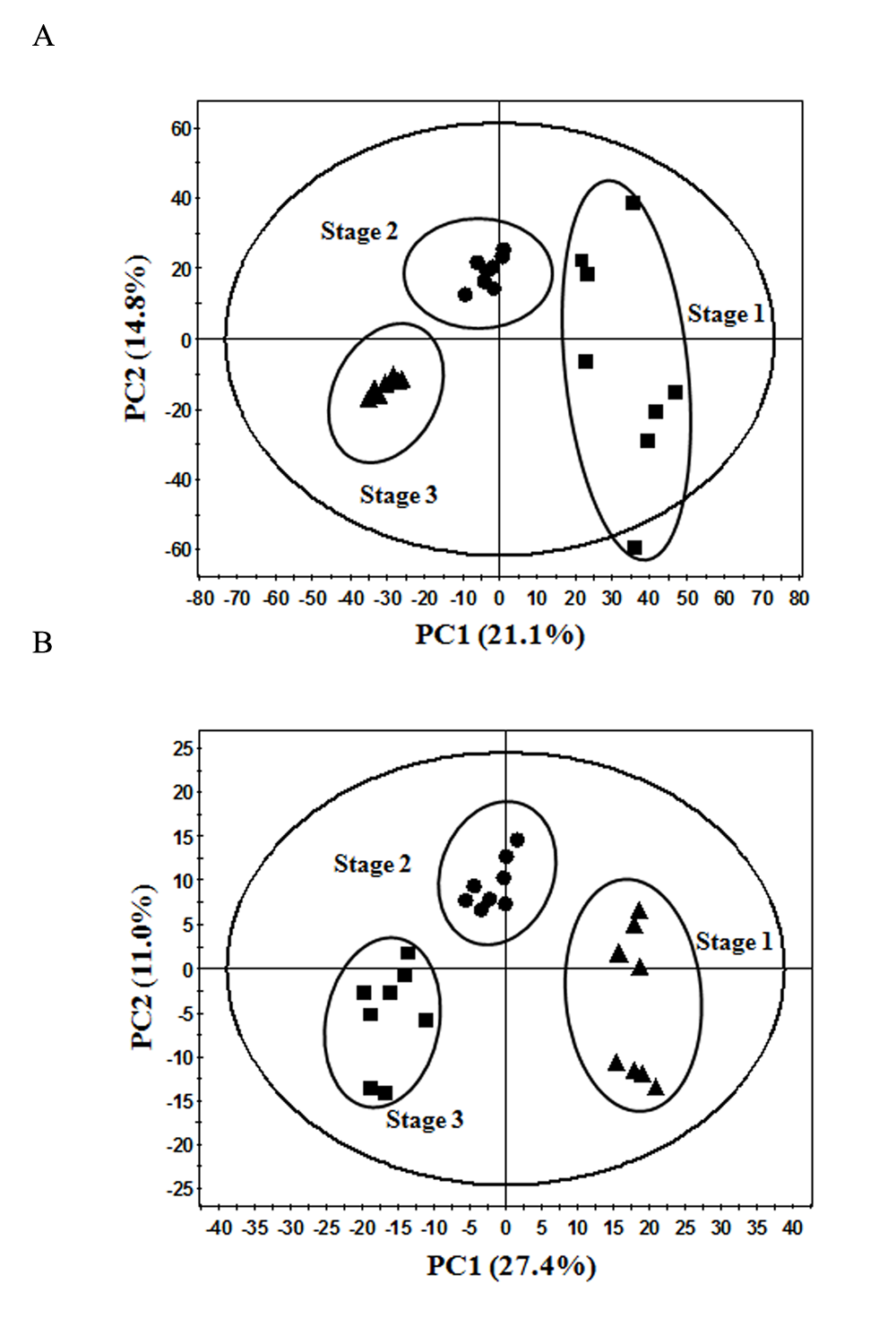

Supplement: Figure S5 — PCA score plots of metabolite profiles in three different ripening stages of Korean black raspberry. The datasets obtained by GC-IT-MS (A) and UPLC-Q-TOF-MS (B) were analyzed by PCA. (TIF) [file pone.0088292.s005.tif]

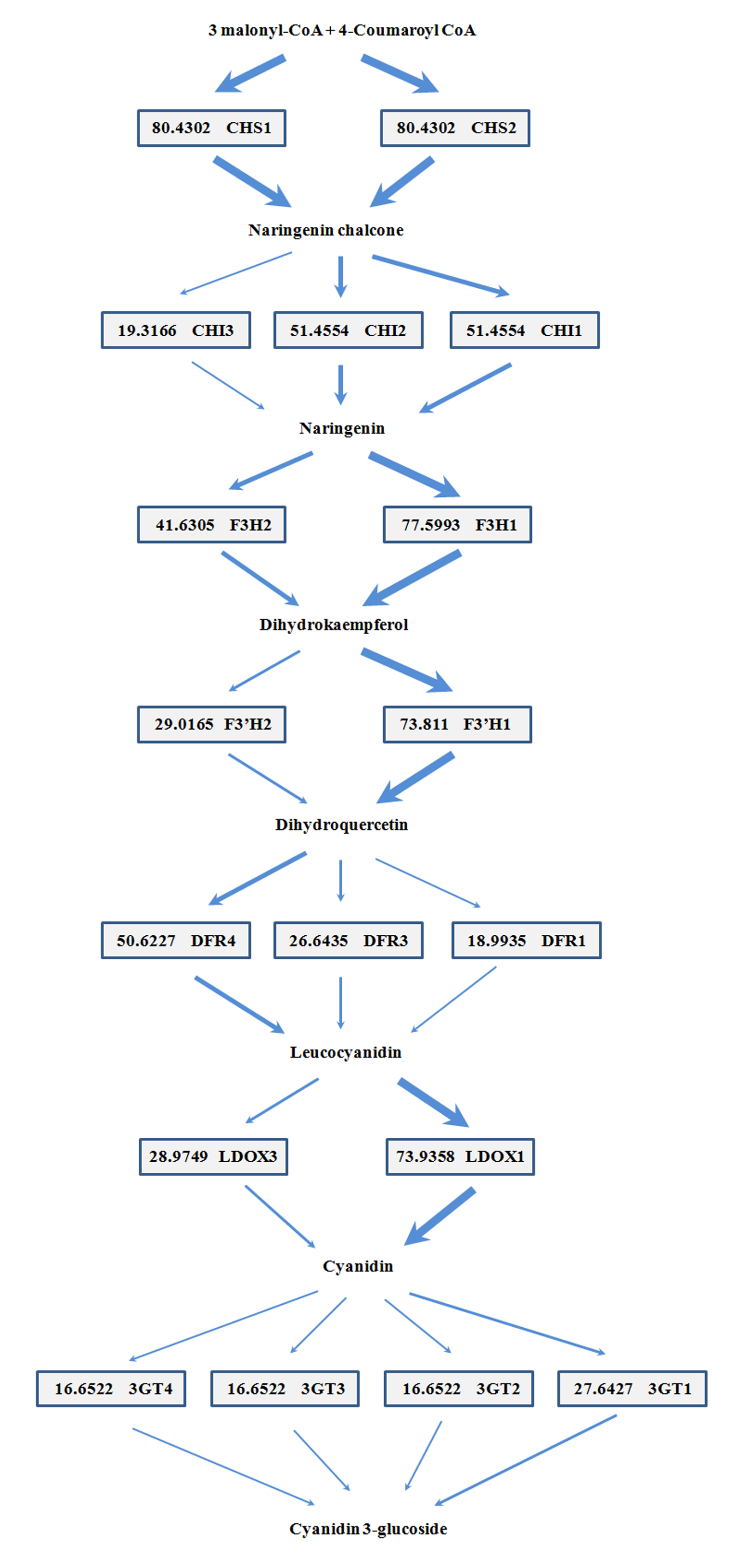

Supplement: Figure S6 — The flux map of anthocyanin biosynthesis during the ripening process. The metabolic flux was analyzed using YANA tool. The numbers along with protein indicate the predicted enzymatic activity of each protein, which was generated by YANA tool. (TIF) [file pone.0088292.s006.tif]

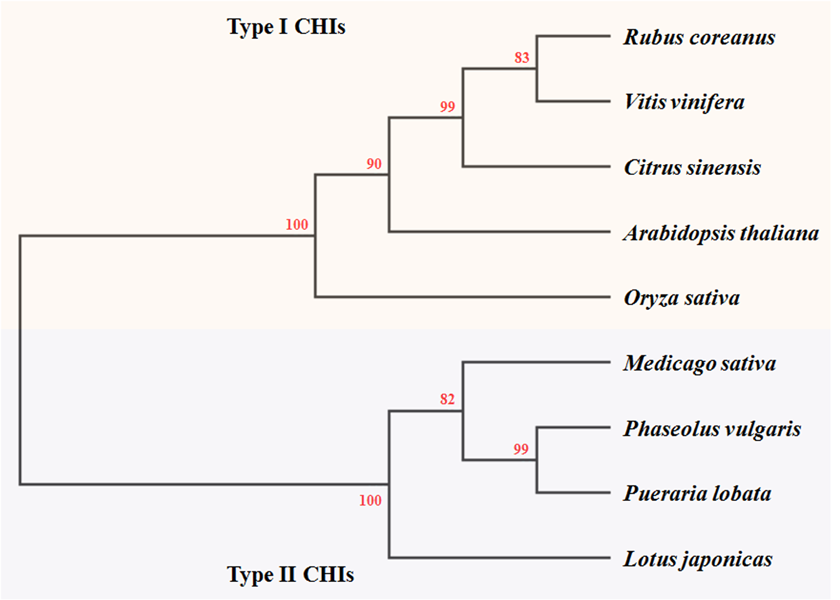

Supplement: Figure S7 — A phylogenetic tree of RcMCHI2 and CHIs from other plant species homology. Amino acid sequences were analyzed using CLUSTALW alignment in PHYLIP format clustal algorithm. Bootstrap values were presented as a percent of 100 resampled trees at each tree node using default settings of the TreeTop-Phylogenetic Tree Prediction (http://www.genebee.msu.su/services/phtreereduced.html). GenBank accession numbers of CHIs from different plant species: Arabidopsis thaliana (At3g55120), Vitis vinifera (CAA53577), Oryza sativa (AF474922), Citrus sinensis (BAA36552), Medicago sativa (P28012), Phaseolus vulgaris (P14298), Lotus japonicas (BAC53983), Pueraria lobata (Q43056). (TIF) [file pone.0088292.s007.tif]
