# Supplementary material for: De-novo RNA Sequencing and Metabolite Profiling to Identify Genes Involved in Anthocyanin Biosynthesis in Korean Black Raspberry (Rubus coreanus Miquel)
Source: PLoS One. 2014 Feb 5;9(2):e88292. doi: 10.1371/journal.pone.0088292 (PMC3914977; doi:10.1371/journal.pone.0088292)
Supplement: Table S1 — Primer sequences for qRT-PCR analysis. (DOC) [file pone.0088292.s008.doc]

**Table S1.** Primer sequences for qRT-PCR analysis.

| Gene name | Primer (5’-3’) |
| --- | --- |
| CHS-1-F | GTTAGAAGCCACGAGGCAC |
| CHS-1-Rev | AGGCCCAAACCCAAATAGT |
| CHS-2-F | GTTAGAGGCCACAAGGAAT |
| CHS-2-Rev | AGGCCCAAATCCAAATAGG |
| CHI-1-F | TGCAAATCAAGTTCACAGCA |
| CHI-1-Rev | TCATCATCCTCTGCAAGCTC |
| CHI-2-F | GAGGGCTCTGGAGATACAGG |
| CHI-2-Rev | CTTCCACTTAACGGCGAGTT |
| CHI-3-F | GTTCGCTCTCCCTAAACGAC |
| CHI-3-Rev | TTTCTCCTCAACCCAATTCC |
| CHI-4-F | CAAATCACCCACTCCTGTTG |
| CHI-4-Rev | AAGGCAGTGGCCAAGTTAAA |
| F3H-1-F | ATCAGAGGCCATGGGTTTAG |
| F3H-1-Rev | TCGGGTAGAAATTGACCACA |
| F3H-2-F | AATCAGCCTATTCGGACTCG |
| F3H-2-Rev | TGATGTCGCCGACTAAACTC |
| DFR-1-F | ATAGGCTCCGGGACTGATAA |
| DFR-1-Rev | TCGACCTTTGTTCATTGCTC |
| DFR-2-F | CACTGGTGTCTTCCACCTTG |
| DFR-2-Rev | ATGCCTAGTGCTCCTTCCAC |
| DFR-3-F | TGTTGGAGGACCATTTCTCA |
| DFR-3-Rev | CCCATTCTTGCATTTACAGC |
| DFR-4-F | CGACCTCTGCCAATCTCATA |
| DFR-4-Rev | GGCAATGTCGTGAATCGTAG |
| F3’H-1-F | AAATCGATGGTGGTGGAGAT |
| F3’H-1-Rev | TCGAATCTCTTGTGCAGCTT |
| F3’H-2-F | ATGGCTATCCCTTCTTCTGG |
| F3’H-2-Rev | TGAGGTTGAGGTTGCCAATA |
| F3’H-3-F | GCAGCTTGCTGATGATCCTA |
| F3’H-3-Rev | TTTAGGAGTTCCGAGATGGC |
| F3'5'H-F | AAGAAATCGAACACGAACCC |
| F3'5'H-Rev | GGAGGTTTGCACCAAGAAAT |
| LDOX-1-F | CACCTTGGAGATTCTGAGCA |
| LDOX-1-Rev | GGATGATCTTCTCCTTGGGA |
| LDOX-2-F | GGACACTGCTGCTCTACCAA |
| LDOX-2-Rev | GTGGTCTAAGCAAGGCATCA |
| LDOX-3-F | TTCTTCCAAATCGTGAACCA |
| LDOX-3-Rev | AAGCCTGCTTCATCTCCATC |
| 3GT-1-F | TCAACACCAAGCAATCCAAT |
| 3GT-1-Rev | AAACATATCCGTCTGGCACA |
| 3GT-2-F | GCATGTTGGCGTTTATGAAG |
| 3GT-2-Rev | CGTTGCAACTACGAACTGCT |
| 3GT-3-F | CGACTTGGATAGCGAGTTGA |
| 3GT-3-Rev | TCTTAGTCAACACCGCCGTA |
| 3GT-4-F | CTACGCAGAGCAGAGGATGA |
| 3GT-4-Rev | TGTATCTCCTCCCTCCCAAC |
| Ubi-F | AAGGTCAAGGGATCTGCAAC |
| Ubi-Rev | CCACAGTCGACTAACCTTTGG |
