# Supplementary material for: De-novo RNA Sequencing and Metabolite Profiling to Identify Genes Involved in Anthocyanin Biosynthesis in Korean Black Raspberry (Rubus coreanus Miquel)
Source: PLoS One. 2014 Feb 5;9(2):e88292. doi: 10.1371/journal.pone.0088292 (PMC3914977; doi:10.1371/journal.pone.0088292)
Supplement: Table S5 — Identification of anthocyanins in Korean black raspberry CHI2 rescued Arabidopsis tt5 mutant. (DOC) [file pone.0088292.s012.doc]

**Table S5.** Identification of anthocyanins in Korean black raspberry *CHI2* rescued *Arabidopsis tt5* mutant.

| **RT** | **UPLC-Q-TOF-MS** | | |  | **LC-IT-MS/MS** | | | | **Predicted metabolites** |
| --- | --- | --- | --- | --- | --- | --- | --- | --- | --- |
|  | **Experimental mass [M-H]-** | **Formula** | **∆ppm** |  | **[M-H]^-^** | **[M+H]*^+^*** | **MS^n^ fragment ions (*m/z*)** | **UV(nm)** |  |
| 3.51 | 609.1458 | C27H29O16 | 0.3 |  | 609 | 611 | 609>447>301 | 270, 535 | delphinidine 3-*O*-rutinoside |
| 3.71 | 593.1510 | C27H29O15 | 0.7 |  | 593 | 595 | 593>447>285 | 270, 533 | cyanidin 3-*O*-rutinoside |
